# Associations between epicardial adipose tissue, subclinical atherosclerosis and high-density lipoprotein composition in type 1 diabetes

**DOI:** 10.1186/s12933-018-0794-9

**Published:** 2018-12-07

**Authors:** Cristina Colom, David Viladés, Montserrat Pérez-Cuellar, Rubén Leta, Andrea Rivas-Urbina, Gemma Carreras, Jordi Ordóñez-Llanos, Antonio Pérez, Jose Luis Sánchez-Quesada

**Affiliations:** 1 Endocrinology and Nutrition Department, Hospital de la Santa Creu i Sant Pau, Barcelona, Spain; 2 Cardiac Imaging Unit, Cardiology Department, Hospital de la Santa Creu i Sant Pau, Barcelona, Spain; 3 Cardiovascular Biochemistry Group, Research Institute of the Hospital de la Santa Creu i Sant Pau (IIB Sant Pau), Barcelona, Spain; 4 CIBER of Diabetes and Metabolic Diseases (CIBERDEM), Barcelona, Spain; 5 Pediatrics Department, Hospital de la Santa Creu i Sant Pau, Barcelona, Spain; 6 Medicine Department, Universitat Autònoma de Barcelona, Barcelona, Spain; 7 Biochemistry and Molecular Biology Department, Universitat Autònoma de Barcelona, Barcelona, Spain

**Keywords:** Type 1 diabetes, Epicardial adipose tissue, Coronary atherosclerosis, High-density lipoprotein, Apolipoproteins

## Abstract

**Background:**

The pathophysiology of cardiovascular complications in people with type 1 diabetes (T1DM) remains unclear. An increase in epicardial adipose tissue (EAT) and alterations in the composition of high-density lipoprotein (HDL) are associated with coronary artery disease, but information on its relationship in T1DM is very limited. Our aim was to determine the association between EAT volume, subclinical atherosclerosis, and HDL composition in type 1 diabetes.

**Methods:**

Seventy-two long-term patients with T1DM without clinical atherosclerosis were analyzed. EAT volume and subclinical atherosclerosis were measured using cardiac computed tomography angiography. EAT was adjusted according to body surface to obtain an EAT index (iEAT). HDL composition was determined.

**Results:**

The mean iEAT was 40.47 ± 22.18 cc/m^2^. The bivariate analysis showed positive associations of the iEAT with gender, age, hypertension, dyslipidemia, smoking, body mass index, waist circumference, insulin dose, and triglyceride (P < 0.05). The iEAT correlated positively with small HDL, increased content of apolipoprotein (apo)A-II and apoC-III, and decreased content of apoE and free cholesterol. Multiple linear regression showed that age, apoA-II content in HDL, and waist circumference were independently associated with the iEAT. Fifty percent of the patients presented subclinical atherosclerotic lesions. These patients had a higher iEAT, and their HDL contained less cholesterol and more apoA-II and lipoprotein-associated phospholipase A2 than patients without subclinical atherosclerosis.

**Conclusion:**

Alterations in the composition of HDL in TIDM are associated with increased iEAT and the presence of subclinical atherosclerosis. We propose that these abnormalities of HDL composition could be useful to identify T1DM patients at highest cardiovascular risk.

**Electronic supplementary material:**

The online version of this article (10.1186/s12933-018-0794-9) contains supplementary material, which is available to authorized users.

## Background

The increase of epicardial adipose tissue (EAT) has been associated with different cardiovascular risk factors such as type 2 diabetes mellitus, hypertension, and obesity, in isolation or as part of the metabolic syndrome (MS) [1–3]. Growing evidence indicates that EAT volume is positively associated with coronary artery disease [4], but the mechanisms involved in this relationship remain largely unknown. Numerous studies have analyzed EAT in type 2 diabetic patients, and its relationship to atherosclerosis development, inflammatory status, endothelial function, and lipoprotein profile has been reported. A major feature of an elevated mass of adipose tissue, including EAT, is an increased non-esterified fatty acid (NEFA) concentration in the blood, the main factor leading to insulin resistance and diabetes. As a consequence of high NEFA levels, diabetic subjects present with increased hepatic apoB synthesis and, moreover, enhanced production of very low density lipoprotein (VLDL), the first step in developing diabetic dyslipidemia [5]. Diabetic dyslipidemia is characterized by increased triglycerides (TG) and apoB and decreased high-density lipoprotein (HDL) concentrations [6]. These quantitative abnormalities are pro-atherogenic per se, but they are also accompanied by qualitative alterations of lipoproteins. HDL from type 2 diabetes has an impaired anti-apoptotic and anti-inflammatory capacity and has partially lost its ability to promote cholesterol efflux from macrophages [7]. The loss of these anti-atherogenic properties of HDL in patients with diabetes is a consequence of proteome remodeling that includes, among other factors, alterations in the content of apolipoproteins (apoA-I, apoA-II, apoC-III, apoE) and enzyme activities with anti-inflammatory properties (lipoprotein associated phospholipase A2-Lp-PLA2-, paraoxonase-1-PON1-) associated with HDL [8]. In turn, the accumulation of epicardial fat is influenced by, among other factors, the abnormalities of the lipid profile, leading to high TG levels in blood, a typical feature of diabetic dyslipidemia.

Cardiovascular complications are the leading cause of death in people with type 1 diabetes (T1DM) [9], but the pathophysiology underlying this relation remains unclear. Although T1DM is mainly characterized by insulin deficiency, other features, such as insulin resistance and metabolic syndrome, are also common pathological derangements contributing to cardiovascular risk in patients with T1DM [10]. In contrast to type 2 diabetes, few studies measuring EAT have been conducted in patients with T1DM. Moreover, in most of these studies, epicardial fat has been inaccurately measured by ultrasound, and only one study has used cardiac computed tomography angiography (CCTA) [11], which is considered the gold standard, along with magnetic resonance imaging (MRI), for the measurement of EAT due to its greater precision and reproducibility [12].

Diabetic dyslipidemia is much less frequent in type 1 than in type 2 diabetes. However, low HDL cholesterol is the most frequent dyslipidemic disorder in poorly controlled subjects with T1DM [13], and some qualitative alterations have been reported in HDL from T1DM, especially in subjects with long-term diabetes. These alterations include decreased antioxidant action, decreased cholesterol efflux capacity, and altered proteome [14, 15]. The loss of these anti-atherogenic properties in HDL probably underlies the premature development of atherosclerosis in T1DM. However, it is not known how EAT affects or is affected by these alterations in HDL and how it contributes to the emergence of atherosclerotic disease in this pathology. Considering the mutual interaction among the accumulation of epicardial fat, the development of atherosclerosis, and the presence of abnormalities in lipoprotein function, the aim of our study was to gain insight into the interrelation among EAT volume, subclinical atherosclerosis, and alterations in the composition of HDL affecting its function in a group of long-term patients with T1DM.

## Materials and methods

### Patients

Subjects with T1DM diagnosed from 1985 to 1994 and regularly followed up in the Endocrinology and Nutrition Department at the Hospital de la Santa Creu i Sant Pau (*n* = 130 patients) were offered participation in the study. The exclusion criteria were the presence of clinical coronary heart disease. Finally, 72 patients underwent CCTA and blood extraction and then attended a medical appointment. Clinical variables were recorded by anamnesis and a review of the patient’s medical history. A physical examination was performed to determine weight, height, body mass index (BMI), waist circumference, and insulin requirements (UI/kg/day). Hypertension was defined as the presence of three or more systolic blood pressures ≥ 140 mmHg, and/or diastolic blood pressure ≥ 90 mmHg, or antihypertensive treatment. Dyslipidemia was defined as the presence of LDL cholesterol levels ≥ 4.2 mmol/L, and/or TG ≥ 2.3 mmol/L, or lipid-lowering treatment. The patients were considered to be smokers if they smoked daily. Overweight was defined as a BMI from 25 to 29.9 kg/m^2^, and obesity was determined if the BMI was greater or equal 30 kg/m^2^. The presence of MS was considered according to the criteria proposed by the International Diabetes Federation (IDF) [16].

### Image analysis

A CCTA exam using a 256-slice CT scanner (Brilliance iCT 256; Philips Healthcare, Amsterdam, the Netherlands) was performed in all participants. A contrast-enhanced scan was performed to assess the atherosclerotic coronary burden and the EAT. This scan was prospectively triggered at 75% of the RR interval using from 100 to 120 kV (120 kV in patients with a BMI > 30 kg/m^2^) if the heart rate was below 65 beats per min (bpm), and retrospectively triggered (helical acquisition) if the heart rate was higher than 65 bpm. The dose of contrast (Xenetix 350; Guerbet, Aulnay-sous-Bois, France) was 0.7–1 ml/kg (range 50–120), followed by a saline flush of 40 ml, both injected at a rate of 5–6 ml/s. Next, CCTA studies were analyzed at an off-line workstation. The method used to calculate the EAT was done with the FDA-cleared OsiriX MD ver. 1.2 software (Pixmeo, Bernex, Switzerland) as follows: first, the upper and lower slice limits of the pericardium were manually defined using an axial view. Next, the EAT was segmented into slices using an algorithm to detect regions of interest with a voxel density from − 150 to − 30 Hounsfield units (corresponding to the adipose tissue). Finally, the procedure was extended to the whole cardiac volume, thus obtaining a contiguous 3-dimensional (3D) volume render (showing EAT volume) quantified in cubic centimeters (cm^3^), as well as indexed to body surface area (EAT index, cm^3^/m^2^).

The atherosclerotic coronary burden in the CCTA exams was performed according to the 16-coronary artery segment model of the American Heart Association (modified 15-segment model, being segment 16 the intermediate branch) [17]. In each coronary artery segment, coronary atherosclerosis was defined as tissue structures 1 mm^2^ that existed either within the coronary artery lumen or adjacent to the coronary artery lumen which could be discriminated from the surrounding pericardial tissue, epicardial fat, or the vessel lumen itself. Coronary atherosclerotic lesions were quantified for stenosis by visual estimation and quantitative coronary angiography (CT-QCA) in CCTA. Stenosis was graded as none, very mild (40% estimated obstruction of coronary luminal diameter), mild (40–49% estimated obstruction of coronary luminal diameter), moderate (50–69% estimated obstruction of coronary luminal diameter), or severe (70–100% estimated obstruction of coronary luminal diameter). The percentage of obstruction of the coronary artery lumen was based on a comparison of the luminal diameter of the segment exhibiting obstruction with the luminal diameter of the most normal-appearing site immediately proximal to the plaque.

### Laboratory determinations

All parameters were determined from plasma obtained in EDTA-containing vacutainer tubes, except PON1 activity that was determined from serum and HbA_1c_ that was measured from total blood. HbA_1c_ was determined by ion-exchange high-performance liquid chromatography (HPLC) (Variant, Bio-Rad laboratories, Hercules, CA, USA).

#### Lipid profile

The lipid profile included TG, total cholesterol, LDL cholesterol, HDL cholesterol, and lipoprotein (a) (Lp (a)). All reagents were from Roche Diagnostics (Bassel, Switzerland) except the Lp (a). The cholesterol of lipoprotein fractions was measured using a direct method to quantify HDL cholesterol (HDL-C plus; Roche Diagnostics, Bassel, Switzerland) or by ultracentrifugation when the TG concentration was higher than 3 mmol/L, according to NECP recommendations. All determinations were performed in a Cobas c501/6000 autoanalyzer (Roche Diagnostics, Bassel, Switzerland). The relative proportion of HDL2/HDL3 was determined using total plasma by non-denaturing (2–16%) polyacrylamide gradient gel electrophoresis (GGE), as described [18].

#### HDL composition

Total HDL was isolated by sequential ultracentrifugation (density range 1.063–1.210 g/ml), using potassium bromide (KBr) gradients. HDL composition was determined by measuring the content of total cholesterol (Roche Diagnostics), TG (Roche Diagnostics), free cholesterol (Wako Chemicals, Richmond, VA, USA) phospholipid (Wako Chemicals), apoA-I (Roche Diagnostics) apoA-II (Kamiya Biomedical Company, Seattle, WA, USA), apoE (Kamiya Biomedical Company) and apoC-III (Kamiya Biomedical Company). Composition data are expressed as a % of HDL total mass. PON-1 activity in serum was measured using phenylacetate as a substrate, as described [19]. Lp-PLA_2_ activity was measured using 2-thio-PAF (Cayman Chemical Company, Ann Arbor, MI, USA) as a substrate, according to the manufacturer’s instructions [20]. To determine the distribution of Lp-PLA_2_ among the lipoprotein fractions, apoB-containing lipoproteins were precipitated from serum using dextran sulfate, as described [21].

### Statistical analysis

To determine the association between qualitative variables and the EAT index (iEAT), we used the contrast t test. To analyze the association between the iEAT and the quantitative variables, the Pearson correlation was used for the variables with a normal distribution, and the Spearman’s rho was used for non-parametric variables. A multivariate analysis of multiple linear regression was performed to determine the variables that independently influence the iEAT. The comparison between groups with or without coronary stenosis was performed with the Student’s *t*-test, except for non-parametric variables that were compared with the Mann–Whitney *U* test. The inter-assay CVs for lipid profile (cholesterol fractions, triglycerides and Lp(a)) are below 7% and those of HDL composition are below 10% for lipids and apoA-I and below 15% for the rest of apolipoproteins. The inter-assay CV of Lp-PLA2 distribution, PON1 activity and HDL2/HDL3 is below 20%.

## Results

### Clinical characteristics of subclinical coronary atherosclerosis, epicardial fat volume, and HDL composition

Table 1 summarizes the clinical characteristics, prevalence of coronary stenosis, and fat volume. All patients were treated with a basal-bolus insulin regimen using injections or an insulin pump. The mean HbA_1c_ following the diagnosis was 55.0 ± 6.1 mmol/mol (7.2 ± 0.8%). Fifty percent of the patients presented some degree of coronary stenosis, although only two of the patients had a coronary stenosis higher than 50%. The mean iEAT of the sample was 40.47 ± 22.18 cc/m^2^ of body surface area, with a median of 38.05 (30.49) cc/m^2^ of body surface area. The results of the lipid profile and HDL composition are shown in Table 2.

**Table 1:** Clinical characteristics, subclinical atherosclerosis, and iEAT volume

Clinical characteristics	Mean ± SD
Male/female	44 (61.1%)/28 (38.9%)
Age (years)	47.1 ± 8.7
Age at diagnosis of diabetes (years)	24.8 ± 8.7
Diabetes duration (years)	22.4 ± 2.1
Insulin dose (UI/kg/day)	0.67 ± 0.22
HbA_1c_ (%)	7.6 ± 1.0
Global BMI/BMI males/BMI females (kg/m^2^)	27.0 ± 4.7/27.9 ± 4.3/25.6 ± 4.9
Waist circumference males/females (cm)	98.3 ± 11.5/87.2 ± 12.6
Overweight/obesity	28 (38.0%)/16 (22.2%)
Hypertension	21 (29.2%)
Dyslipidemia	34 (47.2%)
Statin treatment	31 (43.1%)
Active smoker/ex-smoker	26 (36.1%)/20 (27.8%)
Metabolic syndrome	23 (32%)
Subclinical coronary atherosclerosis	
No lesions	36 patients (50%)
Stenosis < 40%	30 patients (41.7%)
Stenosis 40–50%	4 patients (5.5%)
Stenosis > 50%)	2 patients (2.8%)
Epicardial fat	
iEAT (cm^3^/m^2^)	40.47 ± 22.18

Qualitative variables are expressed as *n* (%) and quantitative variables as mean ± SD

**Table 2:** Lipid profile and HDL composition

Lipid profile	Mean ± SD	Median (interquartile range)
Total cholesterol (mmol/l)	4.79 ± 0.72	4.72 (0.96)
Triglycerides (mmol/l)	1.03 ± 0.91	0.80 (0.48)
LDL cholesterol (mmol/l)	2.84 ± 0.57	2.71 (0.74)
HDL cholesterol (mmol/l)	1.49 ± 0.23	1.49 (0.42)
Lp(a) (mg/l)	313.1 ± 458.1	105.4 (322.3)
HDL-associated parameters		
HDL 2 (%)	54.07 ± 19.29	57.71 (24.93)
HDL 3 (%)	46.85 ± 21.81	42.29 (24.93)
PON1 (µmol/min*ml)	302.51 ± 77.44	297.00 (111.40)
Total Lp-PLA_2_ (µmol/min*ml)	19.13 ± 4.97	18.30 (6.25)
Lp-PLA_2_-HDL (µmol/min*ml)	5.70 ± 1.66	5.40 (1.65)
% Lp-PLA_2_ associated to HDL	31.0 ± 9.6	29.0 (12.0)
HDL free cholesterol (%)	3.33 ± 0.58	3.30 (0.90)
HDL esterified cholesterol (%)	14.21 ± 1.46	14.20 (1.90)
HDL triglycerides (%)	3.77 ± 1.55	3.70 (2.00)
HDL phospholipids (%)	28.70 ± 2.10	28.70 (2.70)
HDL apoA-I (%)	38.10 ± 1.75	38.10 (2.20)
HDL apoA-II (%)	9.60 ± 1.50	9.30 (1.90)
HDL apoE (%)	0.58 ± 0.31	0.60 (0.30)
HDL apoC-III (%)	1.70 ± 0.56	1.70 (0.80)

Data are expressed as mean ± SD and as median (interquartile range)

### Associations of iEAT with clinical variables and HDL composition

iEAT (cc/m^2^ of body surface area) was higher in men than in women, and it was higher in subjects with hypertension, dyslipidemia, active/ex-smoker, and MS than in subjects without these risk factors (Table 3).

**Table 3:** iEAT (cc/m^2^ of body surface area) according to cardiovascular risk factors

	N	Mean ± SD	P-value
Gender			
Male	44	46.9 ± 23.8	0.001
Female	28	30.4 ± 14.9	
Hypertension			
No	51	36.3 ± 19.8	0.011
Yes	21	50.7 ± 24.8	
Dyslipidemia			
No	38	34.3 ± 15.5	0.014
Yes	34	47.4 ± 26.4	
Smoking habit			
Never	26	33.4 ± 19.0	0.043
Active/ex-smoker	46	44.4 ± 23.0	
Metabolic syndrome			
No	49	35.3 ± 18.5	0.003
Yes	23	51.6 ± 25.5	

Data are expressed as mean ± SD

Table 4 shows the correlations found between the iEAT and the quantitative variables. We found no significant correlation between the iEAT and HbA_1c_; instead, we observed positive correlations between the iEAT and age, BMI, waist circumference, insulin dose, and TG. Regarding HDL characteristics, the iEAT correlated positively with the proportion of HDL3 and the content of apoA-II and apoC-III in HDL, and negatively with the content of free cholesterol and apoE. Collectively, these correlations suggest a positive association between EAT accumulation and HDL particles of small size and impaired anti-atherogenic capacity. Furthermore, when patients were classified by the prevalence of HDL2 or HDL3 particles (higher or lower than 50%) the iEAT was increased in those subjects with lower HDL2 proportion (Additional file 1: Figure S1).

**Table 4:** Correlations of iEAT with quantitative variables

	R	P-value
Age (years)	0.430	< 0.001
BMI (kg/m^2^)	0.399	0.001
Insulin dose (UI/kg/day)	0.355	0.002
Waist circumference (cm)	0.559	< 0.001
Mean HbA_1c_	0.176	0.142
Lipid profile		
Total cholesterol (mmol/l)	0.146	0.220
Triglycerides (mmol/l)*	0.333	0.004
LDL cholesterol (mmol/l)*	0.097	0.420
HDL cholesterol (mmol/l)	− 0.040	0.740
Lp(a) (mg/l)*	0.100	0.408
HDL-associated parameters		
HDL 2 (%)	− 0.346	0.004
HDL3 (%)	0.298	0.013
PON1 (µmol/min*ml)	0.145	0.235
Total Lp-PLA_2_ (µmol/min*ml)*	− 0.055	0.652
Lp-PLA_2_ in HDL (µmol/min*ml)*	0.149	0.221
Lp-PLA_2_ in HDL (%)*	0.153	0.208
HDL free cholesterol (%)	− 0.344	0.004
HDL esterified cholesterol (%)	− 0.247	0.041
HDL triglycerides (%)*	0.037	0.763
HDL phospholipids (%)	− 0.159	0.192
HDL apoA-I (%)	0.043	0.724
HDL apoA-II (%)	0.455	< 0.001
HDL apoE (%)	− 0.331	0.005
HDL apoC-III (%)	0.318	0.008

Pearson correlation was used for the variables with a normal distribution and Spearman’s rho for non-parametric variables

* Indicates non-parametric variables

To determine which variables independently explained the increase in the iEAT, a multiple linear regression model was created. First, an analysis of the independence of those variables showing statistical association regarding the iEAT variable in the previous analyses was performed. Among all of the qualitative variables, gender, hypertension, dyslipidemia, smoking, and MS were selected. Among the quantitative variables, those with a correlation higher than 0.3 (see Table 4: age, BMI, insulin dose, waist circumference, total TG, and free cholesterol, as well as apoA-II, apoE, and apoC-III content in HDL) were selected. Table 5 presents the multiple linear regression function obtained from this analysis. The variables selected by the regression function were age, waist circumference and apoA-II content in HDL. The higher the age, the greater the waist circumference, or the greater the content of apoA-II in HDL, the higher the iEAT. The resulting regression function was:

$${\text{iEAT}}\,{ = }\, 0. 4 7 2 { }\,\left( {\text{waist}} \right)\,{ + }\, 0. 9 9 8 { }\,\left( {\text{age}} \right)\,{ + }\, 5. 0 1 0 { }\,\left( {\text{apoA-II HDL mass}} \right) - { 99} . 7 4 5.$$

**Table 5:** Multiple linear regression function for iEAT

	Non-standardized coefficients		Typified coefficients	t	P-value	IC (95%) for B
	B	Typical error				
Waist circumference	0.472	0.196	0.278	2.403	0.019	0.079 to 0.864
Age	0.998	0.254	0.380	3.935	0.000	0.491 to 1.505
ApoA-II in HDL	5.010	1.698	0.329	2.950	0.004	1.616 to 8.403
Constant	− 99.745	18.430		− 5.412	0.000	− 136.574 to − 62.916

To evaluate the adjustment of the regression model to the observed data, different statistics were used, such as the multiple correlation coefficient (*R* = 0.692) and the coefficient of determination (*R*^*2*^ = 0.480). The value of *R*^2^ indicates that slightly less than half (48%) of the variation of the iEAT is explained by the model. The corrected *R*^*2*^ is 0.456. The significance value of *F* (Fisher distribution) = 19.96 with a *P*-value < 0.001 confirms that the model is good

### Comparison between patients with and without subclinical coronary stenosis

Next, we classified patients according to the presence of subclinical atherosclerosis. Only two patients had significant coronary atherosclerotic lesions with stenosis higher than 50%. Therefore, patients were grouped according to the presence of some degree of luminal stenosis (see Table 1). Table 6 shows statistical differences comparing patients with (*n* = 36) and without (*n* = 36) coronary stenosis. Patients with lesions had an average age higher than that of patients without lesions, worse glycemic control, and a higher iEAT. Interestingly, neither BMI nor waist circumference were significantly different between the groups of patients (BMI: 27.2 ± 4.4 kg/m^2^ in patients with lesions versus 26.7 ± 5.0 kg/m^2^ in patients without lesions, *P* = 0.644; waist circumference: 96.0 ± 12.1 cm in patients with lesions versus 92.0 ± 13.8 cm in patients without lesions, *P* = 0.194). This observation points to EAT as a better predictive factor for the development of atherosclerosis than other parameters of general adiposity.

**Table 6:** Comparison of continuous variables between patients with and without coronary stenosis

	With any grade of coronary stenosis (n = 36)	Without coronary stenosis (n = 36)	P-value
HbA_1c_ (%)	7.4 ± 0.8	7.0 ± 0.7	0.047
Age (years)	51.1 ± 9.1	43.2 ± 6.1	< 0.001
iEAT (cm^3^/m^2^)	48.7 ± 21.8	32.3 ± 19.6	0.001
Lipid profile			
Total cholesterol (mmol/l)	4.9 ± 0.8	4.7 ± 0.6	0.199
Triglycerides (mmol/l) *	1.2 ± 1.2	0.8 ± 0.4	0.164
LDL cholesterol (mmol/l) *	2.9 ± 0.6	2.8 ± 0.6	0.525
HDL cholesterol (mmol/l)	1.5 ± 0.3	1.5 ± 0.3	0.988
Lp(a) (mg/l) *	316.5 ± 441.0	305.8 ± 469.8	0.881
HDL-associated parameters			
HDL 2 (%)	53.0 ± 20.1	55.2 ± 18.7	0.657
HDL 3 (%)	47.0 ± 20.1	46.8 ± 23.8	0.701
PON1 (umol/min ml)	316.6 ± 90.3	289.1 ± 59.9	0.156
Total Lp-PLA_2_ (µmol/min ml) *	18.2 ± 4.6	20.1 ± 5.2	0.045
Lp-PLA_2_ in HDL (µmol/min ml)*	5.9 ± 1.6	5.4 ± 1.5	0.190
Lp-PLA_2_ in HDL (%) *	33.9 ± 9.6	28.1 ± 8.9	0.007
HDL total cholesterol (%)	17.1 ± 2.0	18.0 ± 1.4	0.041
HDL esterified cholesterol (%)	13.9 ± 1.7	14.5 ± 1.2	0.060
HDL triglycerides (%)	4.0 ± 1.7	3.5 ± 1.3	0.145
HDL phospholipids (%)	28.6 ± 2.3	28.8 ± 1.9	0.834
HDL apoA-I (%)	37.9 ± 1.8	38.3 ± 1.7	0.260
HDL apoA-II (%)	10.0 ± 1.5	9.2 ± 1.4	0.027
HDL apoE (%)	0.55 ± 0.3	0.6 ± 0.3	0.164
HDL apoC-III (%)	1.8 ± 0.6	1.6 ± 0.5	0.214

Data are expressed as mean ± SD. Comparison between groups with or without coronary stenosis was analyzed with the Student’s *t*-test, except for non-parametric variables that were compared with the Mann–Whitney U test

* Indicates non-parametric variables

Regarding lipid profile, none of the main parameters (total cholesterol, TG, HDL cholesterol, LDL cholesterol, or Lp(a) were significantly different between the groups (Table 6). However, although the level of HDL was similar in both groups, differences in its composition were observed between patients with and without lesions (Table 6). Patients with lesions had HDL particles with less cholesterol and more apoA-II, and they presented a higher activity of Lp-PLA_2_ than HDL particles than the patients without atherosclerotic lesions.

### Effect of statin therapy on iEAT

Lipid-lowering therapy with statins, besides drecreasing cholesterol, has strong effects on lipoprotein composition and properties. Thirty-one (43.1%) patients in the sample used statins (91.2% of patients with dyslipidemia). In order to rule out any confounding effect of statin therapy, we compared subjects with and without statin treatment. We found no difference in lipid profile or in HDL composition between both groups (Table 7). In contrast with lipid profile, thickness of iEAT volume was higher in patients under statin therapy than subjects without statins (47.9 ± 27.2 versus 34.9 ± 15.6, p = 0.013).

**Table 7:** Comparison between patients with and without statin treatment

	With statins treatment (n = 31)	Without statins treatment (n = 41)	P-value
HbA_1c_ (%)	7.9 ± 1.1	7.03 ± 0.9	*0.021*
Age (years)	51.55 ± 8.4	43.8 ± 7.4	*< 0.001*
iEAT (cm^3^/m^2^)	47.9 ± 27.2	34.9 ± 9.0	*0.013*
Lesions (any degree of stenosis)	21 (29.2%)	15 (20.8%)	*0.017*
Lipid profile			
Total cholesterol (mmol/l)	4.6 ± 0.6	4.9 ± 0.8	0.083
Triglycerides (mmol/l)*	1.0 ± 0.6	1.05 ± 1.1	0.320
LDL cholesterol (mmol/l)	2.7 ± 0.5	2.9 ± 0.6	0.067
HDL cholesterol (mmol/l)	1.5 ± 0.3	1.5 ± 0.3	0.796
Lp(a) (mg/l)*	445 ± 618.8	207 ± 218.1	0.148
HDL-associated parameters			
HDL 2 (%)	54.3 ± 17.6	53.8 ± 20.7	0.912
HDL 3 (%)	45.6 ± 17.6	47.8 ± 24.9	0.671
PON1 (μmol/min·ml)	316.1 ± 77.7	291.4 ± 76.5	0.191
Total Lp-PLA_2_ (µmol/min·ml)*	18.2 ± 5.1	19.9 ± 4.8	0.180
Lp-PLA_2_ in HDL (µmol/min·ml)*	5.6 ± 1.4	5.8 ± 1.7	0.491
Lp-PLA_2_ in HDL (%) *	32.3 ± 9.7	29.9 ± 9.6	0.178
HDL total cholesterol (%)	17.6 ± 1.7	17.5 ± 1.8	0.911
HDL esterified cholesterol (%)	14.3 ± 1.5	14.2 ± 1.4	0.795
HDL triglycerides (%)	3.8 ± 1.4	3.7 ± 1.6	0.882
HDL phospholipids (%)	28.3 ± 2.3	29 ± 1.9	0.206
HDL apoA-I (%)	38.1 ± 2.2	38 ± 1.3	0.811
HDL apoA-II (%)	9.9 ± 1.5	9.4 ± 1.4	0.141
HDL apoE (%)	0.5 ± 0.4	0.6 ± 0.2	0.518
HDL apoC-III (%)	1.7 ± 0.6	1.7 ± 0.6	0.703

Italic values indicate significance of p value (p < 0.05)

* Indicates non-parametric values

## Discussion

EAT has become a therapeutic target for preventing cardiovascular injuries associated to obesity and diabetes [22]. EAT volume has been linked to the presence of cardiovascular risk factors in non-diabetic subjects and in individuals with type 2 diabetes [23–28], including male gender, MS, hypertension, dyslipidemia, smoking habits, endothelial dysfunction, BMI, or abdominal circumference. Our data show that these factors are also involved in the accumulation of epicardial fat in T1DM and are in good agreement with previous data reported in other studies conducted in type 1 diabetic patients [11, 29–31]. In concordance with these results, iEAT was 50% higher in patients with moderate or worse coronary stenosis in one or more coronary arteries (48.7 ± 21.8 cm^3^/m^2^) than in patients without apparent lesions (32.3 ± 19.6 cm^3^/m^2^). The mean of the iEAT in our sample was 40.47 ± 22.18 cm^3^/m^2^ of body surface area, with a median of 38.05 (interquartile range 30.49) cc/m^2^ of body surface area. This value is higher than the median detected in a group of healthy individuals (33.3 [range 10.8–96.6] cm^3^/m^2^ of body surface area) used by Shmilovich et al. [32]. These authors placed the threshold value of the iEAT for the prediction of major cardiovascular events at the 95th percentile (68.1 cm^3^/m^2^ of body surface area). Our findings confirm that our type 1 diabetic patients without lesions have similar iEAT levels to those reported by Shmilovich et al. in healthy subjects. We observed that the six patients with a degree of stenosis higher than 50% presented a mean iEAT of 61.8 ± 29.9 cm^3^/m^2^ of body surface area, which is close to the 68.1 cm^3^/m^2^ threshold described by Shmilovich et al. as the 95th percentile for the prediction of cardiovascular events. Thus, our study also suggests that, in T1DM, the accumulation of EAT is closely related to subclinical coronary atherosclerosis.

To the best of our knowledge, this is the first study conducted in patients with T1DM analyzing the relationship between the volume of epicardial fat and the composition of HDL. We found that the volume of EAT was associated with the size of HDL; thus, the higher the iEAT, the higher the proportion of small HDL (HDL3). It is generally accepted that the presence of large HDL particles is associated with low cardiovascular risk, and the prevalence of small-dense HDL is common in MS and type 2 diabetes. In contrast, patients with T1DM usually present large HDL particles (HDL2). Our data suggest that the subgroup of type 1 diabetic patients with small HDL accumulate more EAT than those patients with large HDL particles. This observation concur with the finding by Heier et al. that some T1DM patients present HDL with reduced capacity to promote the cholesterol efflux [15], the first step in the reverse cholesterol transport.

In addition, we found that the content of apoC-III and apoA-II in HDL correlated positively with iEAT, whereas its correlation with apoE content was negative. Collectively, these alterations in HDL, which we found were related with increased iEAT, suggest impairment of its atheroprotective properties. Regarding apoC-III, epidemiological studies have clearly demonstrated that this apolipoprotein is associated with high cardiovascular risk [33]. This is mainly due to the well-established role of apoC-III as an inhibitor of lipoprotein lipase, which results in increasing plasma VLDL levels. However, its role, when associated to HDL, is just beginning to be understood. Subspecies of HDL containing apoC-III are associated with coronary heart disease [34], and increased apoC-III content has been previously reported in HDL from patients with type 2 diabetes and non-diabetic subjects with coronary artery disease [35, 36]. In the latter, it has been suggested that increased apoC-III content compromises the anti-apoptotic activity of HDL [36]; however, the role of apoC-III in epicardial fat accumulation has still not been reported.

An interesting property of apoC-III is its ability to displace apoE from the surface of lipoproteins, which interferes in the clearance of TG-rich lipoproteins [37]. This effect could also be involved in the decrease of apoE in the HDL of type 1 diabetic subjects with a higher EAT volume. Since apoE acts as a ligand for cell receptors for lipoprotein uptake, the decrease of apoE in HDL could affect the clearance of mature HDL particles (HDL2). In fact, apoE distributes preferentially in large HDL particles (HDL2) [8], which agrees with the decreased proportion of HDL2 observed in our patients with higher iEAT values.

Regarding apoA-II, its concentration in blood is positively associated with the elevated plasma levels of free fatty acids, glucose, and insulin [38], which are the parameters related to increased epicardial fat accumulation. In this regard, apoA-II affects the metabolism of large VLDL [39] and promotes the accumulation of visceral fat [40–42]. In the multiple linear regression analysis for the iEAT, the variables selected by the regression function were age, waist circumference, and apoA-II in HDL. The age, with a standardized coefficient of 0.380, is the one that contributes most to the increase of the iEAT, followed by the value of the apoA-II content in HDL (0.329), and then waist circumference (0.278). This analysis again points to the apoA-II content in HDL as a determinant of increased epicardial fat.

When patients were grouped according to the presence or absence of subclinical coronary atherosclerotic lesions, the content of apoA-II in HDL was increased in patients with some degree of coronary stenosis. The content of apoC-III in HDL was also higher in these patients, but did not reach statistical significance. Also, the content of apoE in HDL, albeit decreased, was not statistically different in patients with stenosis. In contrast, we found that the activity of Lp-PLA2 was higher in subjects with coronary stenosis than in those without apparent lesions. This finding was unexpected since the role of HDL-associated Lp-PLA2 is generally considered atheroprotective, and its content is decreased in HDL from type 2 diabetes. This supposes a distinctive characteristic difference between type 1 and type 2 diabetes. However, our data concur with those by Miller et al. [43] who reported that patients with T1DM and coronary disease have increased Lp-PLA2 after adjustment for HDL cholesterol. In contrast, other studies found no difference in Lp-PLA2 content in HDL from type 1 diabetic patients with and without atherosclerosis [44]. Lp-PLA2 is mainly bound to small, dense HDL3, and these particles are increased in subjects with atherosclerosis; thus, it is plausible that, even if we did not reach statistical differences in HDL size between the groups with or without stenosis, the increased content of Lp-PLA2 in HDL from subjects with lesions is related to its association to HDL3. Further studies are necessary to confirm this observation in T1DM with clinical atherosclerosis.

Regarding a putative confounding effect of statin therapy, our observations indicate that even though statin therapy has improved the lipid profile it had no effect on EAT. This does not mean that statins would have no effect on EAT volume, but rather can be explained because patients with statins are older, have a longer history of dyslipidemia, show more coronary lesions and have worse glycemic control than patients without statins. Specifically, age is the main determinant of EAT thickness and must have a strong influence on the accumulation of EAT. Our study was not designed to analyze the effect of statins, and other studies with age-matched patients with and without statins should be conducted to verify any effect of statins on EAT accumulation.

Our study presents other limitations. First, the study focuses only on group of patients with type 1 diabetes with acceptable adherence to treatment followed in a single referral hospital and does not have a control group of patients without diabetes. And second, this is a cross-sectional study whose analysis could not prove clear causality with EAT, a follow-up study may yield more conclusive data.

## Conclusion

In summary, our findings suggest that EAT may contribute to the pathogenesis of coronary atherosclerosis in patients with T1DM. Furthermore, given that remodeling of the HDL proteome is related to both the presence of subclinical atherosclerosis and the excessive accumulation of epicardial adipose tissue, alterations in HDL composition may be a link between epicardial adipose tissue volume and coronary atherosclerosis in T1DM. Specifically, the increased content of apoA-II in HDL seems strongly related to the development of subclinical atherosclerosis and the accumulation of epicardial fat. We propose that patients with type 1 diabetes presenting alterations in HDL composition, despite presenting a good glycemic control, could be at higher risk of developing coronary disease and accumulate more epicardial fat. Therefore, these abnormalities in HDL composition could be a useful tool to identify patients with T1DM with the highest cardiovascular risk.

## Additional file

**Additional file 1: Figure S1.** iEAT of patients grouped according to the proportion of HDL2/HDL3.

## Abbreviations

Apo: apolipoprotein
BHT: butylated hydroxytoluene
BMI: body mass index
CCTA: computed tomography angiography
CT-QCA: quantitative coronary angiography
EAT: epicardial adipose tissue
EDTA: ethylenediaminetetraacetic acid
GGE: native gradient gel electrophoresis
HDL: high density lipoprotein
HPLC: high-performance liquid chromatography
iEAT: epicardial adipose tissue index
LDL: low-density lipoprotein
Lp(a): lipoprotein (a)
Lp-PLA2: lipoprotein-associated phospholipase A2
MRI: magnetic resonance imaging
MS: metabolic syndrome
NEFA: non-esterified fatty acids
PON1: paraoxonase 1
TG: triglyceride
VLDL: very low-density lipoprotein
