# Supplementary material for: Associations between epicardial adipose tissue, subclinical atherosclerosis and high-density lipoprotein composition in type 1 diabetes
Source: Cardiovasc Diabetol. 2018 Dec 7;17:156. doi: 10.1186/s12933-018-0794-9 (PMC6284304; doi:10.1186/s12933-018-0794-9)
Supplement: Supplementary file 1 — Additional file 1: Figure S1. iEAT of patients grouped according to the proportion of HDL2/HDL3. [file 12933_2018_794_MOESM1_ESM.docx]

**Additional Figure S1. iEAT of patients grouped according to the proportion of HDL2/HDL3**
